# Differential Expression Profiles of Cell-to-Matrix-Related Molecules in Adrenal Cortical Tumors: Diagnostic and Prognostic Implications

**DOI:** 10.3390/jpm11050378

**Published:** 2021-05-06

**Authors:** Marco Volante, Ida Rapa, Jasna Metovic, Francesca Napoli, Cristian Tampieri, Eleonora Duregon, Massimo Terzolo, Mauro Papotti

**Affiliations:** 1 Department of Oncology, San Luigi Hospital, University of Turin, 10043 Orbassano, Italy; marco.volante@unito.it (M.V.); ida.rapa@unito.it (I.R.); francesca.napoli@unito.it (F.N.); eleonora.duregon@nih.gov (E.D.); 2 Department of Oncology, Città della Salute e della Scienza, University of Turin, 10126 Turin, Italy; jasna.metovic@unito.it; 3 Department of Medical Sciences, Città della Salute e della Scienza, University of Turin, 10126 Turin, Italy; cristian.tampieri@unito.it; 4 Department of Clinical and Biological Sciences, San Luigi Hospital, University of Turin, 10043 Orbassano, Italy; massimo.terzolo@unito.it

**Keywords:** adrenal cortex, carcinoma, angiogenesis, gene expression, osteopontin, hyaluronan synthase 1

## Abstract

The molecular mechanisms of adrenocortical carcinoma development are incompletely defined. De-regulation of cellular-to-extracellular matrix interactions and angiogenesis appear among mechanisms associated to the malignant phenotype. Our aim was to investigate, employing PCR-based array profiling, 157 molecules involved in cell-to-matrix interactions and angiogenesis in a frozen series of 6 benign and 6 malignant adrenocortical neoplasms, to identify novel pathogenetic markers. In 14 genes, a significant dysregulation was detected in adrenocortical carcinomas as compared to adenomas, most of them being downregulated. Three exceptions—hyaluronan synthase 1 (HAS-1), laminin α3 and osteopontin genes—demonstrated an increased expression in adrenocortical carcinomas of 4.46, 4.23 and 20.32-fold, respectively, and were validated by immunohistochemistry on a series of paraffin-embedded tissues, including 20 adenomas and 73 carcinomas. Osteopontin protein, absent in all adenomas, was expressed in a carcinoma subset (25/73) (*p* = 0.0022). Laminin α3 and HAS-1 were mostly expressed in smooth muscle and endothelial cells of the vascular network of both benign and malignant adrenocortical tumors. HAS-1 was also detected in tumor cells, with a more intense pattern in carcinomas. In this group, strong expression was significantly associated with more favorable clinicopathological features. These data demonstrate that cell-to-matrix interactions are specifically altered in adrenocortical carcinoma and identify osteopontin and HAS-1 as novel potential diagnostic and prognostic biomarkers, respectively, in adrenal cortical tumors.

## 1. Introduction

Adrenocortical carcinoma (ACC) is a rare tumor of the adrenal cortex which accounts for no more than 0.2% of all malignancies. Its differential diagnosis from adrenal cortical adenomas (ACA) has been classically based on several pathological and clinical parameters [1]. None of these is per se indicative of malignancy and is most commonly used in combination in a variety of scoring methods, the Weiss system being the most widely employed. However, such systems are occasionally challenging to apply and/or time consuming despite several implementations introduced in recent years to provide clinically relevant information [2,3,4]. Complementary to histomorphological evaluation, several markers of malignancy have been reported, including p16, p53, topoisomerase II alpha [5,6,7] and Ki-67. In the latter, cut-off values able to discriminate between benign and malignant lesions were set from 2.5% to 4% according to the different authors, whereas in the ACC group, cut-offs ranging from 15 to 20% have been associated with prognosis [7,8,9,10,11,12].

Molecular techniques offer the possibility of a distinction between ACA and ACC and, more importantly, an intrinsic profiling of ACC, as consistently different and specific molecular signatures were identified in these tumors, bearing both diagnostic and prognostic implications [13,14,15,16,17]. At the gene expression level, IGF-2 gene up-regulation represents the most specific molecular signature of ACC, a feature related to genetic alterations at the IGF-2 locus reported in a high proportion of hereditary and sporadic ACC [18,19,20]. IGF-2 immunohistochemical determination has been also proposed as a diagnostic marker of ACC [21]. Among other genes differentially expressed between ACA and ACC, osteopontin and serine threonine kinase 15 were significantly up-regulated (up to 20-fold) in the latter group [17]. In addition, other differential molecular alterations between ACA and ACC were identified within the microRNA or long-non-coding RNA profiles, gene methylation status [22,23,24] or in the expression levels of single biomarkers, including livin/BIRC7 [25], NOTCH pathway [26] and CYP2W1 [27].

However, a detailed comparative investigation of molecules involved in cell-to-matrix interactions and angiogenesis in ACA and ACC is missing. Individual molecules, only have been investigated in adrenocortical tumors, including vascular endothelial growth factor (VEGF), gelatinases, matrix metalloproteinases and others [28,29]. Serum and tissue VEGF protein levels were found significantly increased in malignant tumors [29,30,31]. In addition, matrix metalloproteinases and vascular growth factors, aside from being of interest from a pathogenetic and diagnostic point of view, represent promising targets for targeted therapies [32,33].

By means of a selective PCR-based gene array profiling, our study aimed at analyzing the differential expression of genes involved in cell-to-cell and cell-to-matrix interactions, as well as angiogenic processes, in a series of adrenocortical tumors, with the specific purpose of identifying target genes of potential pathogenetic and diagnostic interest in ACC. The decision to investigate such a family of target genes stemmed from a previous publication from our group on the adverse prognostic role of matrix metalloproteinase type 2 (MMP2) expression in ACC [34], as well as from the reported high expression of osteopontin in adrenocortical tumors [17,35].

## 2. Materials and Methods

### 2.1. PCR-Based Gene Expression Profile

Frozen tissues from 12 adrenocortical tumors, including six ACC and six ACA, were analyzed using a PCR-array based method. Total RNA was extracted using QIAzol lysis Reagent (Qiagen, Tokyo, Japan) according to the manufacturer’s instructions. Clone DNA was transcribed using 500 μg/mL oligodT (Roche, Mannheim, Germany) and 500M-MLV RT (200 U/μL) (Invitrogen, Carlsbad, CA, USA) according to standard protocols.

Expression profiling was detected in duplicate by two gene Array sets for real-time PCR, which includes SYBR Green-optimized primer sets for 12 housekeeping gene probes and a thoroughly researched panel of 157 relevant genes involved in cell-to-cell and cell-to-matrix interactions and angiogenesis (SuperArray Bioscience Corporation, Frederick, MD, USA; codes PAHS-013 and PAHS-024), following the manufacturer’s instructions (Supplementary Table S1). PCR reactions were carried out in a fluorescence-based real-time apparatus (ABI PRISM 7900 Sequence Detection System, Taqman; Applied Biosystems, Foster City, CA, USA), and each 96-well plate was analyzed simultaneously under uniform cycling conditions.

### 2.2. Osteopontin, Laminin α3 and HAS-1 Protein Expression in Adrenocortical Tumors: Case Selection and Immunohistochemistry

Based on the data obtained by gene expression profiling (see below), we aimed at validating the three proteins that resulted in being over expressed in ACC samples. Twenty ACA and 73 ACC tumor tissue samples were analyzed. The former included 42 ACC on tissue micro array (prepared as described elsewhere [36]) and 31 on whole slides. All cases were retrieved from the files at the Pathology Units of AOU San Luigi, Orbassano, and the “Città della Salute e della Scienza”, Turin, hospitals. All adrenocortical neoplasms were independently classified by three of us (MV, ED and MP), according to the Weiss system. For the series of 31 ACC cases analyzed on the whole section, clinical information included sex, age, functional status, tumor weight, tumor size, individual Weiss parameters and overall Weiss score, ENSAT stage, type of tumor recurrence (local and or distant metastases), adjuvant mitotane administration and follow up. All cases were surgical specimens of primary tumor resection. Eighteen patients showed disease progression. Among the seventeen cases with available information on the type of progression, 5 had distant metastases, whereas 12 had local (intra-abdominal) dissemination. Adjuvant mitotane was administered in 23 out of 27 patients with this information available. Before starting the analyses, all samples were anonymized by a staff member of the pathology division not involved in the project, and the study was approved by the Institutional Review Board of the San Luigi Hospital (Protocol AMPRECCO, No. 128/2010).

Five micron-thick paraffin sections were collected onto charged slides and processed for osteopontin (polyclonal antibody, diluted 1:650; Sigma Aldrich, Milano, Italy) and Laminin α3 (1:100, rabbit polyclonal antibody, #PA5-38937, Thermofisher, Waltham, MA, USA) using Ventana BenchMark AutoStainer (Tucson, AZ, USA), whereas hyaluronan synthase 1/HAS-1 (1:250, rabbit polyclonal antibody, #272680, Abcam, Cambridge, UK) was assessed using the Dako Omnis platform (Agilent Technologies, Santa Clara, CA, USA). Appropriate positive and negative controls were included for each immunohistochemical run. Based on the results obtained, immune reactions were scored as follows: osteopontin was scored as negative or positive in the cytoplasm of tumor cells, laminin α3 was scored in vascular structures, only, into a semiquantitative scale based on density of the vascular network (from 0 to 3+), whereas HAS-1 was both scored in vascular structures (same as above) and in tumor cells based on staining intensity (from 0 to 3+).

### 2.3. Statistical Analysis

All data were analyzed with GraphPad Prism 9.0 (GraphPad Softwares, San Diego, CA, USA). A level of *p* < 0.05 was considered statistically significant. Differences in mean gene expression levels between ACC and ACA groups in PCR-array experiments were evaluated by Student’s *t*-test. The differential protein expression of immunohistochemical markers in adrenocortical tumors, as well as the correlation between their expression and clinico-pathological parameters, were estimated by Chi-square test. Univariate disease free and overall survival analysis of positive and negative tumors for each marker and individual clinical and pathological parameters was based on the Kaplan–Meier product limit estimate of disease free and overall survival distribution. Unadjusted differences between survival curves were tested using the LogRank test. Multivariate Cox regression model was used to assess the association of HAS-1 expression and other clinical and pathological variables with disease free and overall survival by means of the SPSS statistical software version 22 (IBM corporation, Armonk, NY, USA).

## 3. Results

### 3.1. Gene Expression Profile of Cell-Matrix Interactive and Angiogenic Proteins in Adrenocortical Carcinomas as Compared to Adenomas

A list of all genes that were significantly differentially regulated between adrenocortical carcinoma and adenoma groups is reported in Table 1. For most of the genes analyzed, a general trend (even for those genes with a fold change not reaching the statistical significance) to down-regulation in ACC as compared to ACA was observed. Exceptions were represented by three genes which were significantly up-regulated in the ACC group. Namely, hyaluronan synthase 1, laminin α3 and osteopontin genes, with a fold change of 4.46, 4.23 and 20.32 times, respectively. Genes just below the statistical significance in their expression fold change between the two groups (with a *p* value >0.05 but <0.1, all down regulated) included members of the chemokine family (CXCL-3, -6 and 9 genes), integrin subunits α8, αν and β3, matrix metalloproteinases and related inhibitors (MMP-2, TIMP-1, -2 and 3), epithelial growth factors (TGFα and EREG-epiregulin genes) and the Tie-2/Tek endothelium-specific receptor tyrosine-kinase.

### 3.2. Osteopontin, Laminin α3 and HAS-1 Protein Expression in Adrenocortical Tumors

**Figure 1:**
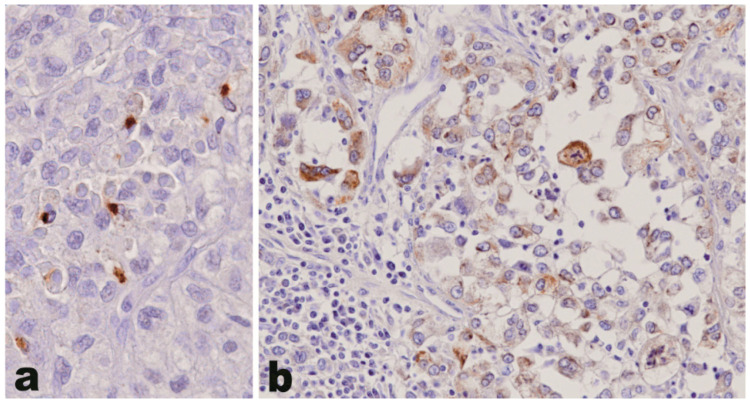
Osteopontin expression in adrenocortical carcinoma. The pattern of staining in adrenocortical carcinoma tumor cells is cytoplasmic, either with a peculiar dot-like paranuclear appearance (**a**) or diffuse within the cytoplasm (**b**). The intratumoral lymphocytes (**b**, bottom left) are un-reactive (original magnification 400×).

By immunohistochemistry, osteopontin was found to be expressed in 25/73 (34%) ACC samples, whereas it was absent in all 20 ACA tested (*p* = 0.0022) (Table 2). The pattern of staining in ACC tumor cells was cytoplasmic, either diffuse or with a peculiar dot-like paranuclear appearance, or a combination of the two (Figure 1) at comparable frequencies. No specific correlation was observed between the presence or absence of staining nor the two staining patterns or the intensity of staining with morphological or clinical features in the 31 ACC patients with clinical information available.

**Figure 2:**
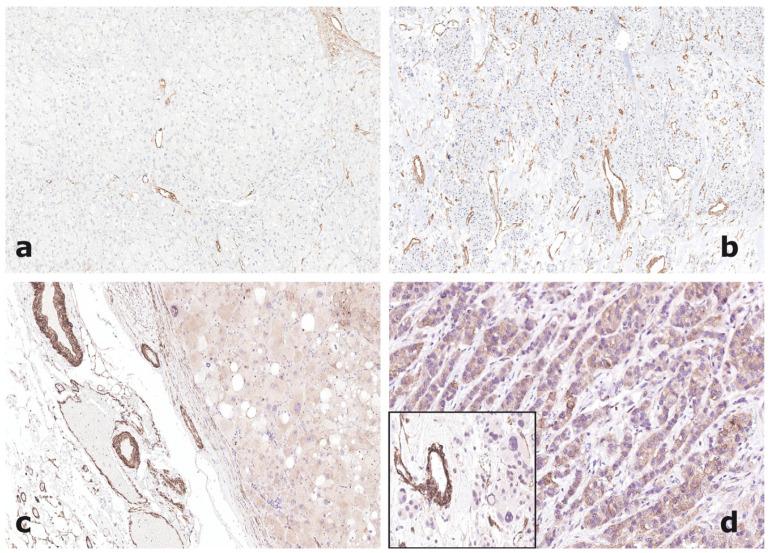
Laminin α3 immunoexpression is restricted to the vascular network of both an adrenocortical adenoma with rare and thin vessels (**a**, 100×) and an adrenocortical carcinoma containing numerous small and medium size vessels (**b**, 100×). Hyaluronan synthase-1 is also strongly expressed by smooth muscle cells of vessel walls in both adrenocortical adenoma (**c**, 100×) and carcinoma (**d** inset, 200×). In addition, tumor cells of adenomas present more frequently a negative or weakly positive pattern of staining (**c**, 100×), while carcinoma cells may show a strong cytoplasmic reactivity (**d**, 100×).

Laminin α3 was expressed both in ACA and ACC, but with a statistically significant increase of density of staining in ACC. The positive staining was observed in both ACA and ACC exclusively in vascular structures, whereas tumor cells were consistently negative in both tumor types (Figure 2a,b). As for osteopontin, no correlation between laminin α3 and clinical or pathological parameters in the series of ACC was observed. HAS-1 did not show a statistically different prevalence of staining in ACA and ACC and was expressed both in vascular structures and in tumor cells, in this latter case with a diffuse cytoplasmic pattern with occasional membrane reinforcement (Figure 2c,d).

HAS-1 was also detectable in the cytoplasm of peritumoral normal adrenal cells, with a more intense staining in cells of the fasciculata zone. HAS-1 positivity in vascular structures was not associated with any of the clinical or pathological variables in the series of 31 ACCs with clinical and pathological annotates.

By contrast, HAS-1 negative expression in tumor cells was significantly associated with features of aggressiveness, such as higher mitotic index, presence of atypical mitoses, and poor disease outcome (Table 3). Negative HAS-1 staining in tumor cells was also more frequently observed in female patients and at lower age of onset.

**Figure 3:**
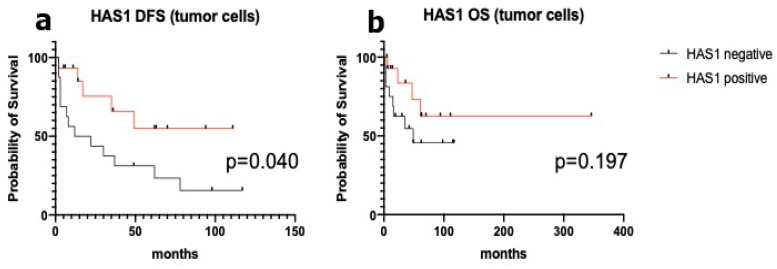
Univariate disease free (**a**) and overall (**b**) survival analysis in HAS-1 tumor cell positive and negative adrenocortical carcinomas.

Disease free (DFS) and overall (OS) survival analyses did not show any significant difference in whether patients were positive or negative for osteopontin or laminin α3. Laminin α3 scores 0–1 had a median DFS and OS of 62 months and “undefined”, respectively, as compared to median DFS and OS of 30 and 61 months, respectively, in cases with 2–3 scores (all *p* values not significant). Osteopontin expression was also not associated with survival, with median DFS of 37 and 22 months and median OS “undefined” and of 61 months, in negative and positive cases, respectively (all *p* values not significant). HAS-1 density of expression in the vascular network was not associated to a specific survival, with median DFS of 22 and 49 months and median OS both “undefined”, in cases with score 0–2 and with score 3, respectively (all *p* values not significant). Interestingly, negative HAS-1 expression was associated with shorter survival (Figure 3). In fact, a trend towards significance was observed for OS, with a 49-month median survival in negative cases as compared to “undefined” in positive cases (Log rank test *p* value 0.197, HR 2.15). Moreover, DFS was significantly different in HAS-1 negative and positive cases, with a median survival of 17 and “undefined”, respectively (Log rank test *p* value 0.040, HR 2.77).

To test the interference of other clinical and pathological factors with HAS-1 in univariate survival analyses, all variables statistically associated with HAS-1 tumor cell positive expression having a *p*-value of <0.2 (see Table 3) were tested in univariate disease free and overall survival analyses. Among those, age, ENSAT stage presence of necrosis and presence of vascular invasion failed to show statistical significance in both disease free and overall survival analyses. By contrast, male sex, size above median (11 cm), mitotic index above median (11 mitotic figures in 50 high power fields) and presence of atypical mitoses were all associated with shorter survival with a *p-*value < 0.2 (*p* values of 0.085, 0.011, 0.088 and 0.078, respectively) and were included in multivariable cox regression analysis together with HAS-1. Independent adverse prognostic impact in terms of disease free survival was retained for male sex and large size (0.036 and 0.028, respectively) only. At overall survival analysis, size above median and mitotic index above median were associated with a *p*-value < 0.2 (*p-*values of 0.082 and 0.084, respectively) and were included in multivariable cox regression analysis together with HAS-1. None of the parameters reached statistical significance, tumor size showing the most significant *p-*value (0.065).

## 4. Discussion

By means of a selective PCR-based gene array approach, we here demonstrated a differential expression of several genes involved in angiogenesis and cell-to-matrix interactions in ACC as compared to ACA. Previous molecular studies in adrenocortical tumors showed peculiar gene expression profiles in ACC as compared to ACA, up-regulation of IGF-2 gene and down-regulation of genes involved in steroidogenesis being the most specific molecular signatures of ACC [17,19,20]. The major advantage of wide gene expression profiling is to generate a large amount of information, thus providing a molecular classification of tumors. On the other hand, data from different studies are not easily comparable and it is sometimes difficult to transpose such complexity of information onto a diagnostic ground. Our approach was to screen a limited number of genes involved in cell-to-matrix interactions and angiogenesis that the previous literature supported to be altered in adrenal cancer [28,29,30,31,34,35], to search for potential pathogenetic or clinically relevant biomarkers. In general, most of the genes that showed a differential expression in ACC as compared to ACA are active regulators of angiogenesis (such as Fibroblast growth factor 2, Hepatocyte growth factor, Prostaglandin-endoperoxide synthase 1, Transforming growth factor, β 1 and Thrombospondin 1). Moreover, several other angiogenesis-regulating genes showed an altered regulation in ACC as compared to ACA, while not reaching statistical significance. Notably, the vast majority of these genes showed a peculiar down regulation. This finding is partially surprising, but it might fit with the consistent down-regulation of several growth factor receptors described by Giordano and co-workers in ACC [17] and it might represent either the consequence of the different vascular network in ACA vs. ACC or the consequence of decreased tissue expression. Due to the heterogeneous biological functions of these molecules, it is difficult, however, to hypothesize the biological meaning of our findings, in the lack of functional experiments that represents a major limitation of our study. Moreover, among the genes that showed no differential or slightly lower expression in ACC, the various matrix metalloproteinases, their specific inhibitors, and VEGF isoforms should be mentioned since their related proteins have been found to be over-expressed in ACC tumor cells [29,30,31]. In this respect, the absence of a differential expression of the corresponding genes between ACA and ACC in our series may more probably reflect a balance between the integrity of the vascular network and neo-angiogenic capabilities in the two tumor types, the equilibrium being more shifted to the former in ACA and to the latter in ACC [31].

An altered expression of laminin isoforms was observed in the current series of ACC, with a higher expression of the α3 and lower expression of α2 and α5 laminin isoforms, as opposed to ACA. This observation is interesting since α2 and α5 laminin isoforms are consistently expressed in the basal membrane of the normal adult adrenal cortex, whereas α3 isoform is virtually absent [37], and might be related to structural alterations of the basal membrane in ACC tissue [38]. In fact, the reduced expression of α2 and α5 laminin isoforms may be correlated with the morphological finding of reticulin framework disruption in carcinomas [38], while overexpression of laminin α3 might be related to the higher expression levels of this isoform observed in the dense intratumoral vascular network of ACC.

With regard to other molecules than laminin α3 having an increased expression in ACC, osteopontin had the highest fold difference, a finding in agreement with previous gene array data [17] and with the report of Weisman and co-workers [35]. It was therefore further analyzed in a large series of adrenocortical tumors by means of immunohistochemistry to test its tissue localization pattern. Osteopontin expression was restricted to a fraction of malignant cases, being absent in all ACAs, at variance with the findings of Weismann and co-workers who observed a heterogeneous expression of OPN in both ACA and ACC, with a staining often restricted to single cells or small clusters. In our series, however, osteopontin provided a specificity of 100% but a sensitivity of 34% only, thus limiting its value as a diagnostic tool in the differential diagnosis between benign and malignant adrenocortical tumors.

Osteopontin is a secreted phosphoprotein that binds multiple integrins including αvβ1, αvβ3, and αvβ5 [35] and is involved in several cellular processes, such as cell attachment, spreading and migration, homing of lymphocytes and other hematopoietic cells and vascular remodeling [39,40].

Moreover, osteopontin has been demonstrated to be over-expressed in many human tumors, including carcinomas of the breast [41], lung [42], ovary [43], stomach [44], liver [45], and prostate [46] as well as mesothelioma [47], and it was proposed as a potential prognostic marker in many of these neoplasms. In particular, osteopontin expression is induced by hypoxia and its prognostic role in solid tumors is partially explained by its interplay with other hypoxia-related molecules and angiogenic factors, at least in head and neck and prostate cancer [48,49].

Interestingly, in different cellular models osteopontin up-regulates the expression of hyaluronan synthases. In breast cancer cells, osteopontin has been shown to upregulate HAS-2, another enzyme involved in the synthesis of hyaluronan, a major component of the extracellular matrix, with a role in several cellular functions, including cell proliferation and migration [50]. Moreover, in osteoarthritic-derived chondrocytes, osteopontin has been shown to promote the expression of the HAS-1 gene [51]. In the present series, HAS-1 was identified by immunohistochemistry in the smooth muscle and endothelial cells of blood vessels of both benign and malignant adrenocortical tumors, with no differences except for the denser vascular network in ACC. In addition, HAS-1 was also detected in the tumor cells of a fraction of ACC (42%), but only occasionally and weakly in adenoma tumor cells (although this difference was not statistically significant). This finding is not in agreement with gene expression data on the frozen series and claims that HAS-1 has no role as a diagnostic marker in the differential diagnosis between ACA and ACC. However, the lack of a direct correlation between HAS-1 gene and protein data in ACC vs. ACA might be explained by the wide heterogeneity of protein distribution and intensity in blood vessels, as well as in tumor cells. In fact, although with no statistical significance comparing the different subgroups, both in tissue blood vessels and in tumor cells, ACC showed a higher rate of HAS-1 intense (3+ score) staining, which might explain the overexpression observed at the gene level. More intriguingly, HAS-1 expression in ACC tumor cells was associated with a lower mitotic rate, a lower prevalence of atypical mitotic figures and a better outcome. The prognostic significance of HAS-1 in cancers is equivocal and has been studied either in cancer cells or in cancer stroma, including cancer-associated fibroblasts [52,53,54]. However, in agreement with our findings, a negative prognostic role of HAS-1 downregulation in cancer cells has been described in lung cancer [55] and melanoma [56]. Survival analysis in our study has several limitations, including the small sample size and incomplete data on progression that could not allow a disease-specific survival, but overall survival only. The association of negative HAS-1 tumor cell expression with shorter disease free survival, that showed statistical significance in univariate analysis, could not be validated as an independent factor in multivariate analysis due to the possible interference with other significant negative prognostic factors such as tumor size. Nevertheless, even though our data need to be confirmed in a larger series, the prognostic impact of HAS-1 expression claim a role for the interplay between cancer cells and the stromal environment as a relevant field of investigation in ACC, which is at variance with several other cancer models, has not yet been explored in detail.

## 5. Conclusions

In conclusion, the present paper showed that ACC is associated to an altered expression of several genes involved in angiogenesis and cell-to-cell/cell-to-matrix interactions, possibly as the result of unbalanced vasculature and neo-angiogenic properties. Among these, osteopontin is selectively expressed in ACC tumor cells and represents a potential diagnostic marker, although with a remarkably low sensitivity, whereas negative HAS-1 expression in tumor cells characterizes a subset of ACC with a more aggressive clinical outcome.

## Figures and Tables

**Table 1:** Adrenocortical carcinoma versus adenoma significant fold differences in gene expression among 157 molecules investigated.

Gene Name	Description	*t*-Test (*p-*Value)	Fold Up/Down Regulation
BFGF/FGFB	Fibroblast growth factor 2	0.0484	−5.66
DHAND2/Hed	Heart and neural crest derivatives expressed 2	0.0234	−13.61
HAS1	Hyaluronan synthase 1	0.0287	4.46
F-TCF/HGFB	Hepatocyte growth factor	0.0489	−9.02
IL-1/IL1-BETA	Interleukin 1, β	0.0448	−8.00
LAMA	Laminin, α_2_	0.0391	−16.00
E170/LAMNA	Laminin, α_3_	0.0409	4.23
KIAA1907	Laminin, α_5_	0.0280	−3.86
COX1/COX3	Prostaglandin-endoperoxide synthase 1 (cyclooxygenase)	0.0442	−5.20
BNSP/BSPI	osteopontin, bone sialoprotein I	0.0450	20.32
CLEVER1/FEEL1	Stabilin 1	0.0215	−7.45
CED/DPD1	Transforming growth factor, β 1	0.0365	−5.53
THBS/TSP	Thrombospondin 1	0.0456	−10.82
DIF/TNF-alpha	Tumor necrosis factor (TNF superfamily, member 2)	0.0270	−11.66

**Table 2:** Immunoexpression of Laminin α3, HAS-1 and osteopontin in a series of 73 adrenocortical carcinomas and 20 adrenocortical adenomas.

	IHC Semiquantitative Score	* ACC (#73)	§ ACA (#20)	*p* Value
Laminin α3 (vascular network)	0	13 (17.8%)	3 (15%)	0.04
	1+	20 (27.4%)	11 (55%)	
	2+	23 (31.5%)	6 (30%)	
	3+	17 (23.3%)	0	
HAS-1 (tumor cells)	0	42 (57.5%)	13 (65%)	0.55
	1+	17 (23.3%)	5 (25%)	
	2+	7 (9.6%)	2 (10%)	
	3+	7 (9.6%)	0	
HAS-1 (vascular network)	0	12 (16.4%)	2 (10%)	0.51
	1+	21 (28.8%)	9 (45%)	
	2+	18 (24.7%)	5 (25%)	
	3+	22 (30.1%)	4 (20%)	
Osteopontin (tumor cells)	negative	48 (65.8%)	20 (100%)	0.0022
	positive	25 (34.2%)	0	

Abbreviations: IHC: immunohistochemistry; * ACC: adrenocortical carcinoma, 31 cases on whole section and 42 on TMA; § ACA: adrenocortical adenoma (all on whole section).

**Table 3:** Major clinico-pathological features according to HAS-1 positivity in tumor cells (#31 cases on whole section).

Parameter		HAS-1 Positive	HAS-1 Negative	*p* Value
Sex	M	8	6	0.02
	F	7	10	
Age	<46 *	3	10	0.02
	≥46	12	6	
Functional status	Nonfunctioning	8	10	0.62
	Functioning ^†^	7	6	
Tumor weight (g)	<275 *	10	9	0.55
	≥275	5	7	
Tumor size (cm)	<11 *	10	6	0.10
	≥11	5	10	
Mitoses	<8 *	10	4	0.02
	≥8	5	12	
Atypical mitoses	absent	11	6	0.045
	present	4	10	
Necrosis	absent	4	1	0.12
	present	11	15	
Venous invasion	absent	2	6	0.12
	present	13	10	
Sinusoid invasion	absent	8	9	0.87
	present	7	7	
Capsular invasion	absent	8	8	0.85
	present	7	8	
Nuclear atypia	absent	3	4	0.74
	present	12	12	
Weiss score	3–6	8	7	0.59
	7–9	7	9	
ENSAT stage	I	4	0	0.068
	II	10	13	
	III	1	3	
Mitotane treatment (4 cases missing)	no	3	1	0.60
	yes	12	11	
Status	NED	10	3	0.0069
	AWD/DOD	5	13	
Median DFS	months	not reached	49	0.040
Median OS	months	not reached	17	0.197

Legend. DFS: disease free survival; OS: overall survival; *: median value; ^†^: functioning tumors were defined according to the presence of an evident clinical syndrome and included among the hormones produced cortisol, aldosterone, and androgens; NED: no evidence of disease; AWD: alive with disease; DOD: dead of disease.

## Data Availability

Not applicable.

## Supplementary Materials

The following are available online at https://www.mdpi.com/article/10.3390/jpm11050378/s1, Table S1: Panel of 157 genes involved in cell-to-cell and cell-to-matrix interactions and angiogenesis tested in the present study.
